# Tissue Specific Localization of Pectin–Ca^2+^ Cross-Linkages and Pectin Methyl-Esterification during Fruit Ripening in Tomato (*Solanum lycopersicum*)

**DOI:** 10.1371/journal.pone.0078949

**Published:** 2013-11-13

**Authors:** Hiromi Hyodo, Azusa Terao, Jun Furukawa, Naoya Sakamoto, Hisayoshi Yurimoto, Shinobu Satoh, Hiroaki Iwai

**Affiliations:** 1 University of Tsukuba, Faculty of Life and Environmental Sciences, Tsukuba, Ibaraki, Japan; 2 Hokkaido University, Creative Research Institution (CRIS), Sapporo, Hokkaido, Japan; 3 Hokkaido University, Natural History Sciences, Sapporo, Hokkaido, Japan; Institute for Plant Protection (IPP), CNR, Italy

## Abstract

Fruit ripening is one of the developmental processes accompanying seed development. The tomato is a well-known model for studying fruit ripening and development, and the disassembly of primary cell walls and the middle lamella, such as through pectin de-methylesterified by pectin methylesterase (PE) and depolymerization by polygalacturonase (PG), is generally accepted to be one of the major changes that occur during ripening. Although many reports of the changes in pectin during tomato fruit ripening are focused on the relation to softening of the pericarp or the Blossom-end rot by calcium (Ca^2+^) deficiency disorder, the changes in pectin structure and localization in each tissues during tomato fruit ripening is not well known. In this study, to elucidate the tissue-specific role of pectin during fruit development and ripening, we examined gene expression, the enzymatic activities involved in pectin synthesis and depolymerisation in fruit using biochemical and immunohistochemical analyses, and uronic acids and calcium (Ca)-bound pectin were determined by secondary ion-microprobe mass spectrometry. These results show that changes in pectin properties during fruit development and ripening have tissue-specific patterns. In particular, differential control of pectin methyl-esterification occurs in each tissue. Variations in the cell walls of the pericarp are quite different from that of locular tissues. The Ca-binding pectin and hairy pectin in skin cell layers are important for intercellular and tissue–tissue adhesion. Maintenance of the globular form and softening of tomato fruit may be regulated by the arrangement of pectin structures in each tissue.

## Introduction

Fruit ripening is a developmental process accompanying seed development. In fleshy fruits, this involves many physiological processes, including the production of nutrients and aromatic compounds, changes in colour and softening of the pericarp, which evolved to attract animals and promote seed dispersal [1]. The molecular pathways that underlie many ripening-related phenomena have been characterised, including the modification of fruit nutritional and organoleptic status as well as the role of ethylene in ripening, such as changes in colour, flavour and shelf life [2], [3], [4], [5], [6], [7], [8]. However, the critical molecular determinants of fruit firmness and softening are not well known.

Fruit softening is a prominent character in fleshy or climacteric fruits. For more than 40 years, many studies have targeted the mechanism of fruit softening, much of it using tomato fruits as a model system to study fleshy fruit development and ripening. A decrease in fruit firmness occurs due to dissolution of the primary cell wall and middle lamella, resulting in a reduction in intercellular adhesion, depolymerization and solubilisation of hemicellulosic and pectic cell wall polysaccharides [9]. These events are accompanied by increased expression of various cell wall-degradation enzymes. For example, polygalacturonase (PG)-catalysed depolymerization of pectin in the wall and middle lamella was long believed to be the principal process underlying fruit softening in tomatoes [9]. However, suppressing PG by constitutive expression of antisense PG transgenes driven by the cauliflower mosaic virus 35S promoter, which yielded transgenic fruits retaining only 0.5–1% of the wild-type level of PG enzyme activity, did not affect overall fruit ripening and softening [10]. Similarly, suppressing the expression of several other ripening-related cell wall modification proteins, such as pectinesterase (PE)-catalysed de-methyl-esterification of pectin, in transgenic tomato fruits has generally resulted in minimal effects on fruit softening or texture [9], [11], [12]. On the other hand, suppression of β-galactosidase activity early in ripening significantly reduces fruit softening [13]. Although small effects on fruit softening can be achieved by individual gene knockdowns [9], [14], substantial changes in fruit texture are likely to require the simultaneous modulation of multiple pectin degradation-related genes.

Although pectin depolymerization is well known to be a characteristic change in tomato fruit ripening, it does not directly affect fruit softening and firmness. And recently, it was suggested pectin de-methlesterification by PE effects to BER in tomato fruit (*Solanum lycopersicum*) that believed to be a calcium (Ca^2+^) deficiency disorder. [15] The function and the changes in pectin structure and localization in each tissue during tomato fruit ripening is not well known. Although many reports on tomato fruit ripening are focused on the relation between pectin degradation and softening of whole fruits or the pericarp, changes in pectin content and/or composition during fruit ripening may be unique between tissues.

**Figure 1:**
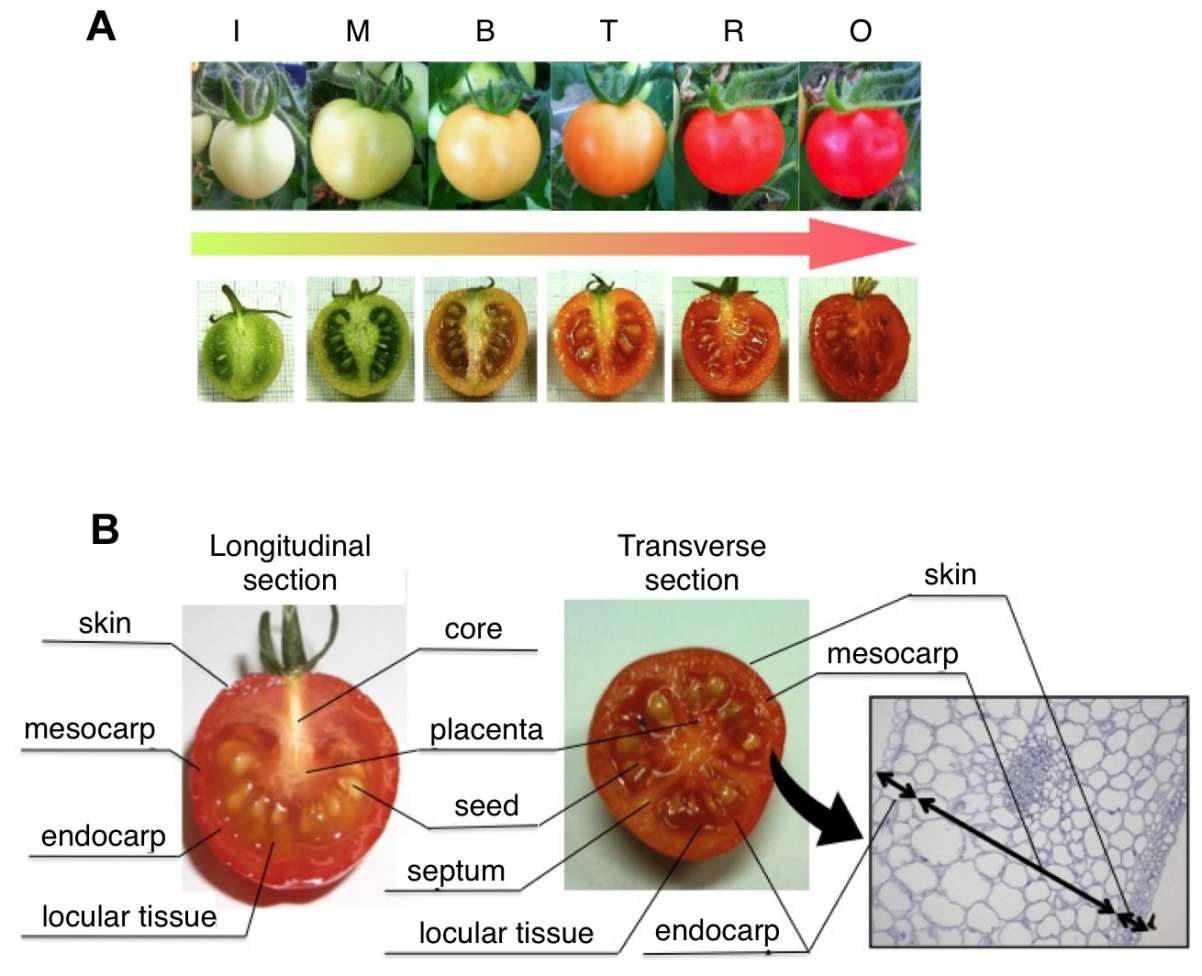
Preparation for tissue-specific analysis. A, The fruit ripening stages of cv. Micro Tom. The six stages included immature green (I), mature green (M), breaker (B), turning (T), red ripe (R) and overripe (O). B, The fruit tissues of cv. Micro Tom. The eight tissues included skin, mesocarp, endocarp, septum, locular tissue, seed, placenta and core. These were separated by hand-sectioning.

In this study, to elucidate the tissue-specific role of pectin during fruit development and ripening, we examined the expression of pectin biosynthesis/depolymerization genes, targeting glycosyltransferase-1-like gene (GAUT1-like), pectin esterase (PE2) and PG2. The enzymatic activity, content and composition of pectin uronic acids involved in pectin biosynthesis and depolymerization in tomato fruit tissues were examined. In the previous reports, observations of fruits were only in pericarp, because red ripe fruit has liquefied locular tissues, which was quite difficult to keep in the microscopic sample (Fig. 1). In this reports, we have succeeded in observation of longitudinal section of red ripe tomato fruit by devise method of microscopic fix technic. Here, we report successful preparation of longitudinal sections through red ripe tomato fruit enabled by a novel microscopic fixation technique. We tried immunohistochemical analyses of uronic acids and calcium (Ca)-bound pectin localization in whole tomato fruit.

## Materials and Methods

### Plant Material

Tomatoes (*Solanum lycopersicum* cv 'Micro Tom') were grown inside a cultivation chamber (TOMY CL-301) under 16 h light and 8 h dark at 26°C and 22°C, respectively, and a light intensity of approximately 100 µmol m^–2^ s^–1^. Tomato fruits at the corresponding developmental stages were also collected: I (<1 cm length; 15 days after pollination (dap), M (30 dap), B (35 dap), T (37 dap), R (45 dap) and OR (55 dap); Figure 1).

### RNA Expression Analysis

Total RNA extractions were performed using a Qiagen RNeasy Mini Kit (Qiagen, Valencia, CA, USA) with subsequent DNase treatment to remove any contaminating DNA. RNA was quantified by spectroscopy. Quantitative RT-PCR assays were performed using the TaKaRa ExTaq Kit (TaKaRa Bio, Otsu, Japan). The PCR conditions were as follows: step 1, 94°C for 3 min; step 2, 98°C for 10 s; step 3, 50°C for 15 s and 72°C for 1 min (steps 2–4; x times). The primer and probe pairs for each gene assayed are given in Table S1. The expression level of RNAs was visually analysed on ethidium bromide-stained gels.

### Determination of Pectin Methyl-esterase Activity

Pectin methyl-esterase (PE) activity was assayed with a continuous spectrophotometric method according to Hagerman and Austin (1986) [16] in extracts of the enzyme obtained from tomato fruit tissues. The corresponding control sample contained enzyme extracts obtained from untreated tissue. Activity measurements were conducted at 20°C and pH 7.5 in a cuvette containing 2 ml pectin, 0.15 ml bromothymol blue (BTB) and 0.55 ml distilled water. Addition of 0.3 ml enzyme extract started the reaction and the residual enzyme activity was immediately assayed. The change in absorbance at 620 nm was recorded for 10 min in a UV/VIS spectrophotometer (Perkin-Elmer, Waltham, MA, USA). The activity values reported are an average of three independent measurements. The assay was calibrated daily at room temperature and activity expressed as units of absorbance (–0.0001) per second.

### Determination of PG Activity

PG activity was assayed by determining the liberated reducing end products following the method of Milner and Avigad [17], in which polygalacturonic acid is used as a standard. The reaction mixture contained 100 µl of enzyme extract and 300 µl of Milner–Avigad copper reagent boiled for 10 min. After rapid cooling, we added 100 µl of Nelson reagent as a color former. After stirring, 800 µl of DW was added, followed by incubation at room temperature for 30 min. We recorded the absorbance at 600 nm using a UV/VIS spectrophotometer (Perkin-Elmer, Waltham, MA). The activity values are reported as averages of three independent measurements. Activity is expressed as change in the content of isolated uronic acid per unit time.

### Extraction and Analysis of Cell Wall Polysaccharides

Tomato samples were frozen in liquid nitrogen. The frozen tissue was powdered in a mortar in liquid nitrogen and the resulting powder washed in 80% EtOH. The supernatant was removed after centrifugation for 5 min at 15,000 × *g*. The pellet was washed three times with water, three times with methanol:chloroform (MC  =  1:1) and three times with acetone. A drop of phenol:acetic acid:water (PAW  =  2:1:1) was added to the pellet and mixed. Two drops of MC were added to the sample and then washed with acetone. This process was repeated three times and the sample was then dried at room temperature for over 1 h. Starch was removed by digestion with amylase (2 unit/ml amylase; Wako Pure Chemical Industries, Osaka, Japan) in 50 mM acetate buffer at 37°C for 3 h. After reaction, the samples were centrifuged and the residues were washed three times with water, MC and acetone. After washing, the samples were air-dried for over 12 h. AIRs were used as the cell wall material. A total of 2 mg of AIR was hydrolysed with 2 M trifluoroacetic acid (TFA) at 121°C for 2 h. After hydrolysis, the samples were centrifuged at 15,000 × *g* for 5 min. The supernatant was the TFA-soluble fraction. The pellets were hydrolysed with 72% H_2_SO_4_ at room temperature for 2 h and then diluted to 4% H_2_SO_4_ and boiled for 1 h. The H_2_SO_4_ solutions were neutralised with Ba(OH)_2_. Sugar in TFA-soluble and -insoluble fractions was treated with methanol:hydrogen chloride and the resulting methyl glycosides were converted and analysed by gas–liquid chromatography (GC-2010; Shimadzu, Kyoto, Japan). Sugar content in TFA-soluble and TFA-insoluble fractions was determined using the phenol sulphuric acid method.

### Determination of the Pectin Methyl Ester Content

The methyl ester group was determined quantitatively by an enzymatic method involving an alcohol oxidase/formaldehyde dehydrogenase system. For hydrolysis of methyl esters bound to pectin, 0.1 m KOH (100 µl) was added to the pectin solution (100 µg/100 µl), followed by standing for 1 h at room temperature. The methanol released was determined. The reaction mixture, composed of 100 mM glutathione (60 µl), 100 mM NAD+ (60 µl), alcohol oxidase (1 unit) and FADH (2 units) in 0.2 M potassium phosphate buffer (pH 7.5) in a total volume of 2.9 ml, was placed in screw-cap tubes. Aliquots (100 µl) of methanol standards (0.5–10 µg) or the pectin hydrolysates containing 50 pg of galacturonic acid were added to the tubes. The tubes were incubated at 25°C for 30 min. The methanol content was calculated using e  =  6.2 × 10^3 ^mol^–1^ cm^–1^ for NADH at 340 nm. The degree of methyl esterification (DE) was expressed as the molar per cent of methyl ester groups per d-galacturonic acid residues.

### Tissue Section and Light Microscopy

Tomato fruit pericarp samples were cut by hand-sectioning. These samples were fixed in 2.5% paraformaldehyde in 0.025 mM phosphate-buffered saline (PBS) and evacuated using a vacuum pump for 12 h. Fixed samples were dehydrated through the following series of ethanol concentrations: 30%, 50%, 70%, 80% and 90% for 20 min each and then 95% and 100% twice for 30 min. Ethanol in dehydrated samples was exchanged for Technovit 7100 resin (Heraeus Kulzer, Wehrheim, Germany) through the following series of Technovit 7100:ethanol: 1:4, 2:3, 3:2, 4:1 each for 30 min and then 100% Technovit for 30 min and 12 h. Samples were then solidified in Technovit 7100 resin following the manufacturer's protocol. Embedded samples were cut into 5-µm sections using a microtome with a glass knife. The sections were stained with 1% Toluidine Blue and 1% Ruthenium Red solution for 10 min, washed with water and then observed under a microscope (×40). Whole tomato fruit samples were cut in half by hand sectioning to prevent the liquefied locular tissues from leaking out from fruit samples during fixation and ethanol dehydration treatments. These samples were fixed in 2.5% paraformaldehyde in 0.025 mM PBS and evacuated with a vacuum pump for 24 h. Fixed samples were dehydrated through the following sequence of ethanol concentrations: 30%, 50%, 70%, 80%, 90% and 95%; dehydrations were repeated thrice for 20 min in each ethanol concentration. Sections were then immersed in 100% ethanol thrice for 30 min. Although not all tissues in the half-cut fruit samples were completely fixed, about 3–4 mm wide thicknesses of tissue adjacent to the hand-cut surfaces were (including liquefied locular tissues). Accordingly, we removed about 3–4 mm of tissue from either side of the hand-cut surfaces for embedding in resin. Ethanol-dehydrated samples were immersed in the following sequence of Technovit 7100 resin concentrations (in ethanol): 50% for 6 h, followed by 100% for 6 h and 12 h. Samples were then solidified in Technovit 7100 resin following the manufacturer's protocol. Embedded samples were cut into 10-μm-thick sections using a microtome with a tungsten knife. The sections were stained with 1% Toluidine Blue and 1% Ruthenium Red solution for 10 min, washed with water, and then observed under a light microscope (×40).

### Immunohistochemical Analysis

A series of monoclonal rat IgG antibodies to Homogalacturonan / LM19 and LM20 was purchased from PlantProbes (Leeds, UK; www.plantprobes.net) and a TSA kit with HRP-conjugated secondary antibody and Alexa Fluor 488 tyramide were purchased from Invitrogen (Carlsbad, CA, USA; cat. noT20912). Immunohistochemistry using the set of monoclonal antibodies followed the manufacturer’s instructions. The sections were put under PBS prior to labelling and 100 µl of the following reagents were dropped onto the sections in order: quenchin buffer (to quench endogenous peroxidase activity), 1% blocking reagent and primary antibody diluted in 1% blocking reagent (1:30), each time incubated at room temperature for 1 h. The sections were washed three times with PBS, then incubated in 100 µl of HRP conjugate diluted in 1% blocking reagent (1:100) for 1 h, washed (3× PBS) and incubated in 100 µl of tyramide working solution [tyramide stock solution diluted in amplification buffer/0.0015% hydrogen peroxide (H_2_O_2_); 1∶100) for 10 min at room temperature and washed three times with PBS followed by Distilled Water (DW), twice. The sections were mounted in DW and observed under a fluorescence microscope.

### Determination of Ca Content

Fruit tissues and AIRs were homogenised with a mortar and pestle, and the samples (300 mg fresh weight and AIRs) were pre-digested overnight in a solution of 40% nitric acid (HNO_3_) and 10% H_2_O_2_. Subsequently, samples were digested in concentrated HNO_3_ at 140°C. To measure metal concentrations, we first filtered digested solutions diluted with Milli-Q through 0.45-μm membrane filters (Millipore, Billerica, MA, USA). After dilution with 0.1 N HNO_3_, We determined the Ca content by inductively coupled plasma atomic emission spectroscopy (ICP-AES; Optima 7300 DV; Perkin-Elmer) at the Chemical Analysis Centre, University of Tsukuba. To calculate the concentrations of these elements, we obtained standard solutions from Wako Pure Chemical Industries.

### Ion Microscope Analysis

Samples were solidified in Technovit 7100 resin following the manufacturer's protocol. Embedded samples were stained with 1% Ruthenium Red solution for 5 min and washed with water. The samples were coated with a 30-nm layer of gold to avoid the accumulation of charge due to the primary beam of the ion microscope and electron microprobe. Ion microscope analyses were conducted at Hokkaido University using a modified Cameca ims-1270 ion microscope with a SCAPS ion imager to undertake two complementary ion beam techniques [18]. A O^–^ primary beam of 23 keV was homogeneously irradiated on the sample surface of approximately 300×300 µm^2^ with a beam current of 10∼40 nA [19]. Secondary ion images of ^102^Ru^+^, ^63^Cu^+^, ^40^Ca^+^ and ^39^K^+^ were obtained with the exposure times of 100 s, 50 s, 50 s and 25 s for each isotope images.

## Result

### Analysis of Pectin Biosynthesis and Depolymerization-related Gene Expression in Tomato Fruit Tissues during Ripening

**Figure 2:**
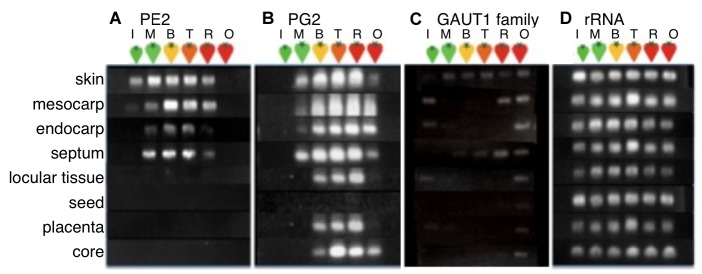
Pectin biosynthesis/degradation-related gene expression patterns differed among tissues. Gene expression was analysed by RT-PCR. A, PE2, pectin methyl-esterase 2 (25 cycles); B, PG2, polygalacturonase 2 (25 cycles); C, GAUT1 family *Arabidopsis* pectin homogalacturonan galacturonosyltransferase-like gene family (25 cycles); D, rRNA, as a control (20 cycles). Expression levels were compared to *rRNA* in the same assay. The eight tissues analyzed in these assays included skin, mesocarp, endocarp, septum, locular tissue, seed, placenta, and core. Ripening stages were the following: I, immature green; M, mature green; B, breaker; T, turning; R, red ripe; O, overripe.

To compare ripening-related cell wall pectin metabolism between tomato fruit tissues, the expression of genes encoding proteins involved in pectin biosynthesis, modification and depolymerization, including glycosyltransferase-1 (GAUT1-like), pectin methyesterase2 (PE2) ([20], [21], [22]; GenBank accession no. X07910), and polygaracturonase 2 (PG2) ([23],[24]; GenBank accession no. X14074.1) was examined by reverse transcription-polymerase chain reaction (RT-PCR) analysis (Fig. 2). GAUT1 was the first successfully identified pectin biosynthetic enzyme, homogalacturonan (HG) α-1, 4-GalA transferase identified in *Arabidopsis thaliana* [25]. GAUT1 was predicted to be a Type 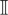 membrane protein, with a single N-terminal transmembrane helix and a main globular domain inside the Golgi lumen. In tomatoes, pectin biosynthetic enzyme was not identified, and so we examined a homologous genes of GAUT1. Homology search of the SOL database (http://solgenomics.net/) and Mibase (http://www.pgb.kazusa.or.jp/mibase/) found a GAUT1 homologous genes (GAUT1-like family) in tomatoes. These tomato GAUT1-like family have high similarity to homologous to *Arabidopsis* GAUT1 protein based on the nucleic acids of tomato unigenes registered with the SOL Genomics Network (SGN), including a conserved glycosyltransferase family 8 domain (Figure S1). PE2 and PG2 are known ripening-related pectin modification and depolymerization enzymes. These enzymes are believed to be expressed and active in fruits. PE catalyses the de-methyl-esterification of pectin HG polymers and assists in the depolymerization of HG by PG. PE also promotes Calcium (Ca)-binding between HG polymers. PE expression is generally thought to increase gradually during fruit ripening, and PG expression to increase gradually in analyses of whole tomato fruits or fruit pericarp [26]. However, the expression of these genes in each of the tomato fruit tissues has not been demonstrated. We analysed expression by RT-PCR and found differences in expression between tissues.

Figure 2 shows the expression patterns of four fruit-associated tomato pectin biosynthesis/degradation-related genes during fruit development and ripening. The expression of tomato rRNA was also evaluated in the same analysis as a loading control. The expression patterns in different fruit stages were generally similar. For example, PE2 and PG2 were expressed at high levels in ripening fruit pericarp, as previously reported [26], [27], [28], [29]. Remarkably, PE2 expression was not detected in tissues surrounding seeds, such as locular tissue, seed, placenta, and core (Fig. 2A). PG2 expression in tissues surrounding seeds was delayed compared with other tissues (Fig. 2B). The expression pattern of the GAUT1-like gene gradually increased during fruit ripening (Fig. 2C).Pectin content may differentially change between tissues during ripening, and thus enzymatic assays of PE and PG activity and quantitative determination of cell wall and pectin contents were performed.

### Determination of PE Activity and Degree of Pectin Methyl-esterification (DE) in Tomato Fruit Tissues during Ripening

**Figure 3:**
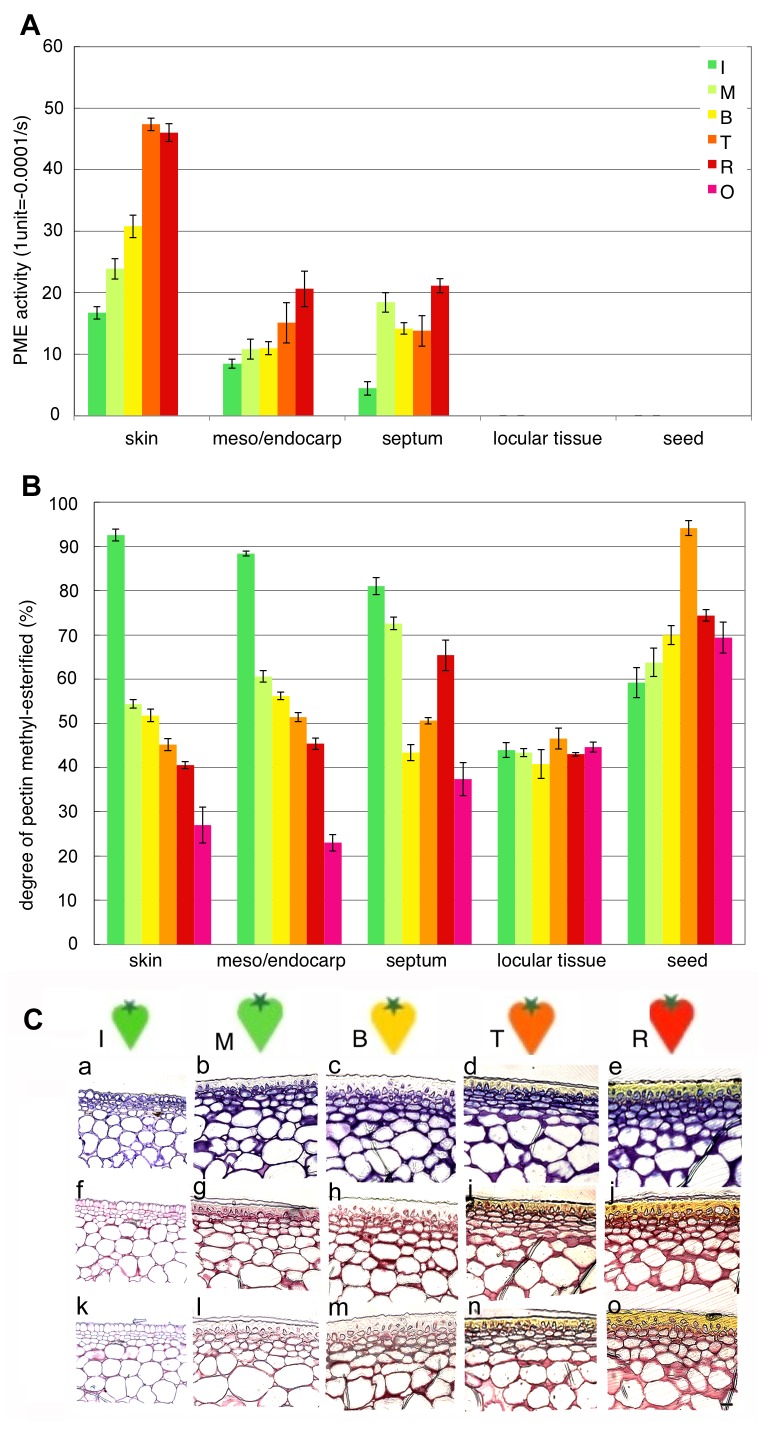
PE activity and a decreasing degree of pectin methyl-esterification were specific to pericarp tissues. A, PE activity. Total protein in the cell wall was extracted from each fruit tissue and assayed for PE activity. One unit means a decrease in OD_620_ per second. B, Degree of pectin methyl-esterification. The pectin fraction was extracted from fruit tissues for analysis. The five tissues analysed in these assays included skin, mesocarp/endocarp, septum, locular tissue and seed. Ripening stage: I, immature green; M, mature green; B, breaker; T, turning; R, red ripe; O, overripe. ±SD of three independent replicates. C, Pectin localisation between the skin and the mesocarp. Light microscopy images with Toluidine Blue (a-e) as a control, Ruthenium Red after NaOH treatment for 5 min (f-j) staining of pectin and Ruthenium Red (k-o) staining of de-methyl-esterified pectin. Ripening stage: I, immature green; M, mature green; B, breaker; T, turning; R, red ripe.

Pectin methyl-esterase (PE) activity was assayed by a continuous spectrophotometric method following Hagerman and Austin (1986) [16] with extracts of the enzyme obtained from five tomato fruit tissues (skin, mesocarp/endocarp, septum, locular tissue and seed). PE activity was remarkably high in the skin and gradually increased during ripening in skin, mesocarp/endocarp and septum. In locular tissue and seed, PE activity was not detected (Fig. 3A). Analysis of the degree of pectin methyl-esterification showed that the DE of the pericarp decreased during ripening, while DE did not change during ripening in seed surrounding tissues where it was maintained at about 50% (Fig. 3B).

### Determination of the Ca Content in Tomato Fruit Tissues during Ripening

**Figure 4:**
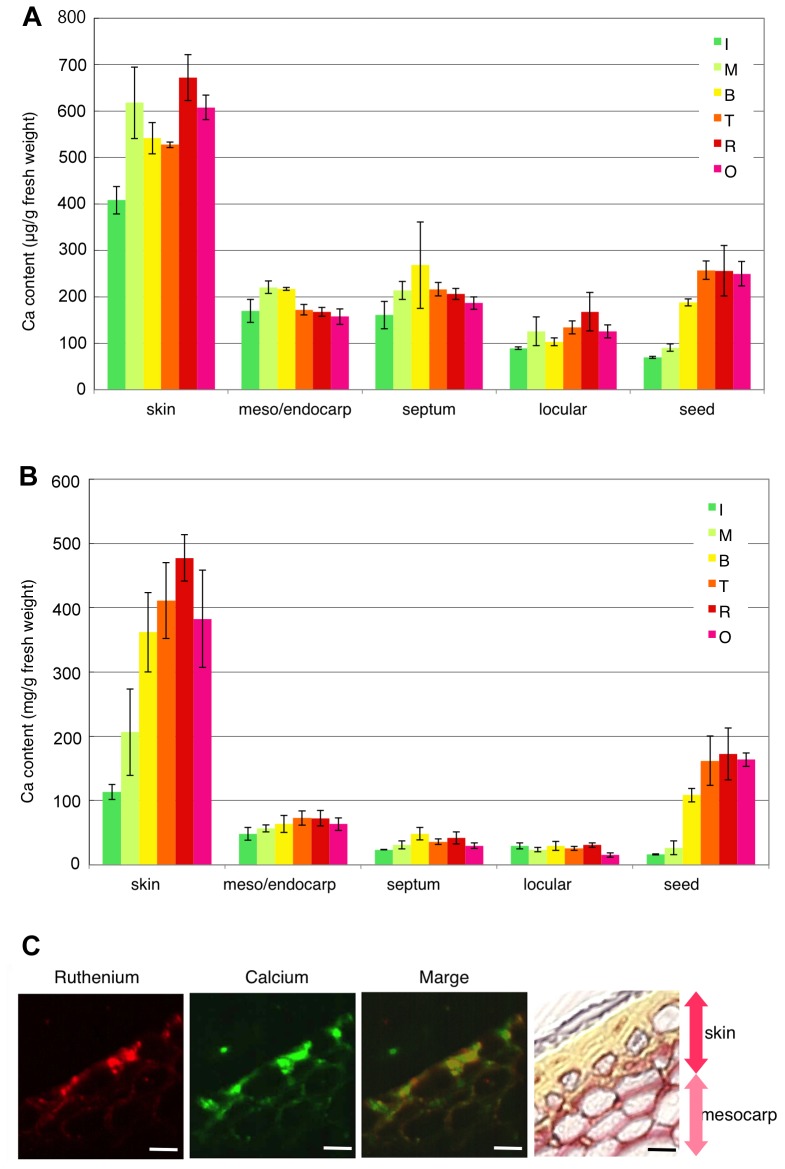
Ca-bound pectin was plentiful in the boundary cell layers between the skin and mesocarp. A, Ca content in tomato fruit tissues. B, Ca content in the tomato fruit cell wall. The alcohol-insoluble residue fraction was extracted from fruit tissues for analysis. Ca content in each fraction was determined by inductively coupled plasma atomic emission spectroscopy (ICP-AES). C, Ca determination between the skin and mesocarp. Quantitative imaging by secondary ion-microprobe mass spectrometry (SIMS). Ruthenium: showing ruthenium, which binds with pectin. Calcium: showing calcium content. Merge: showing overlap of ruthenium (pectin) and calcium.

Total Ca content in alcohol-insoluble residues (AIR) samples from each tissue was determined by inductively coupled plasma-atomic emission spectroscopy (ICP-AES). Total Ca content was very high in the skin compared with other tissues. In skin, locular tissue and seed Ca content increased gradually during ripening (Fig. 4A). In contrast, in the septum, Ca content remained steady and decreased slightly in the mesocarp/endocarp. Similarly, Ca content in the AIR was very high in the skin and increased at the breaker stage (Fig. 4B). In mesocarp/endocarp, Ca content increased after the B stage and in the septum and locular tissue, Ca increased and decreased during ripening, respectively (Fig. 4B). Ca in the AIR sample could be the result of cell wall (pectin)-binding calcium. Determination of Ca by secondary ion-microprobe mass spectrometry (SIMS) suggested similar results (Fig. 4C). Direct observations of Ca^2+^-pectin localisations in fruit by SIMS are reported here for the first time. Ca was high in the skin, especially in cell layers between the skin and mesocarp. Ca localised with pectin, which was stained by Ruthenium Red (Fig. 4C).

### Determination of PG Activity in Tomato Fruit Tissues during Ripening

**Figure 5:**
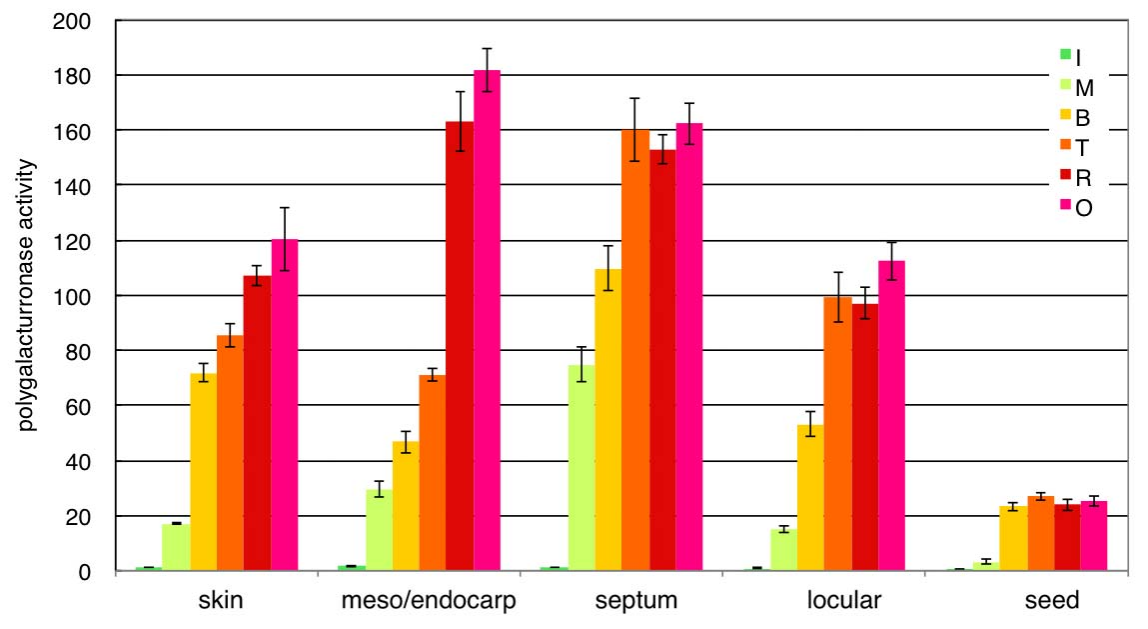
PG activity affects fruit softening in pericarp tissues at the late ripening stage. PG activity was determined by the Milner-Avigad method (Milner and Avigad, 1967). Total protein in the cell wall was extracted from each tomato fruit tissue and assayed for PG activity. The five tissues analysed in this assay included skin, mesocarp/endocarp, septum, locular tissue and seed. Ripening stage: M, mature green; B, breaker; T, turning. ±SD of three independent replicates.

To determine the PG activity in tomato fruit tissues, cell wall protein extracts from five tomato fruit tissues (skin, mesocarp/endocarp, septum, locular tissue and seed) at three ripening stages (M-T) were assayed for their ability to degrade polygalacturonic acid *in vitro* (Fig. 5). Figures show that protein extracts isolated from pericarp tissues possessed high PG activity at the M stage, while seed surrounding tissues had weak activity at the M stage and activity gradually increased from the B stage. Remarkably high PG activity was observed in the septum.

### Biochemical Analysis of the Cell Walls of Fruit Tissues during Ripening

**Figure 6:**
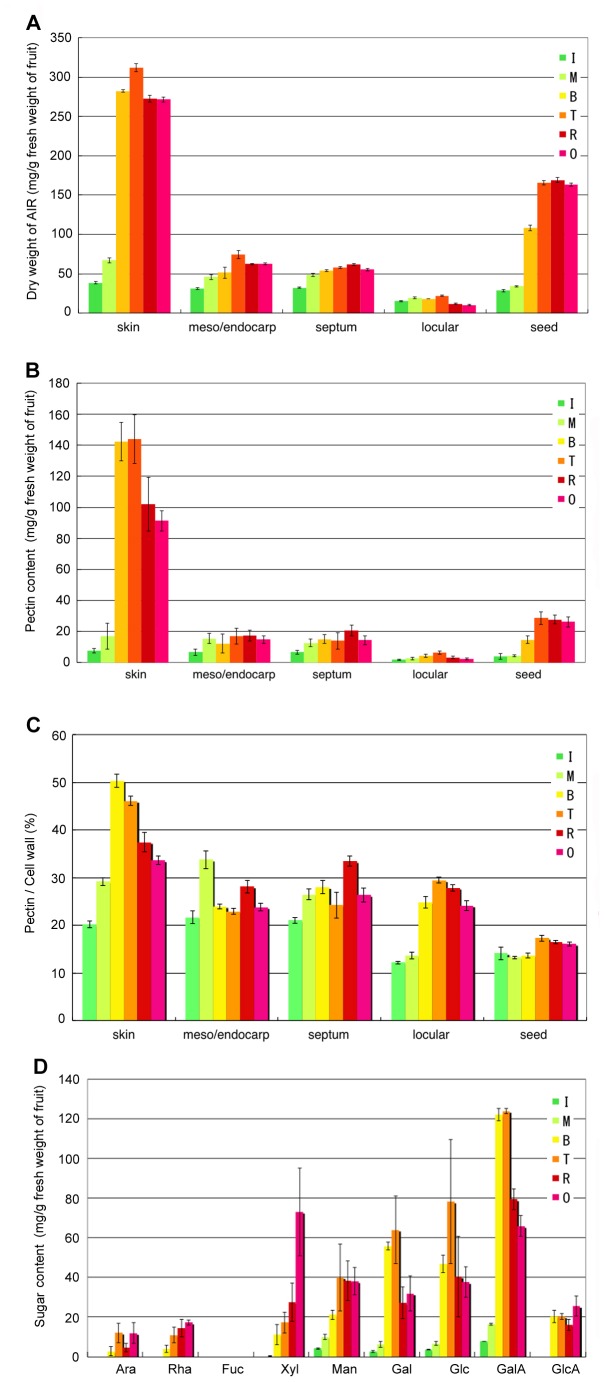
Changes in the cell wall and pectin content differed in fruit tissues during ripening. A, Dry weight of the alcohol-insoluble residue (AIR) per 1 g fresh weight from each fruit tissue. B, Pectin content per 1 g fresh weight from each fruit tissue. C, Relative pectin content in the cell wall. The five tissues analysed in this assay included skin, mesocarp/endocarp, septum, locular tissue and seed. D, Suger content in pectin fraction extracted from skin cell wall. Ripening stage: I, immature green; M, mature green; B, breaker; T, turning; R, red ripe; O, overripe. ±SD of three independent replicates.

Changes in the amount of total cell wall material (on a fresh weight basis) and of uronic acids followed tissue-specific and typical ripening-related trends (Fig. 6A–C). In all tissues, total cell wall content increased from the I to T stages accompanying fruit and seed development. The pectin content (uronic acids) also increased in the cell wall. In the skin, pectin content decreased remarkably from the T to R stages (Fig. 6B). This suggests that a pectin degradation-related enzyme, like PE, affects pectin content during ripening. Sugar composition analysis of the pectin fraction indicated that most tomato fruit pectins were HG; pectin sugar composition diversified in the skin during fruit ripening (Fig. 6D).

### Distribution of Methyl-esterified Pectin, Non-methyl-esterified Pectin in Tomato Fruit Tissues during Ripening

**Figure 7:**
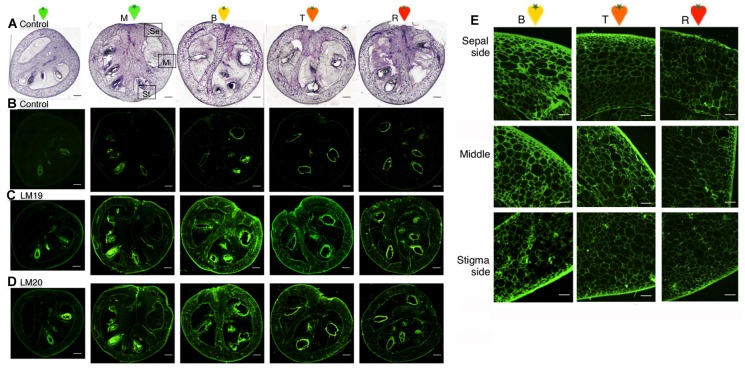
Immunolocalization of Homogalacturonan (HG) epitopes in tomato fruit longitudinal section of fruit. A, Light microscopy images with Toluidine Blue as a control. B, Negative control. C, Immunolabeled with LM19, which labelled de-methyl-esterified HG. D, Immunolabeled with LM20, which labelled methyl-esterified HG. Ripening stage: I, immature green; M, mature green; B, breaker; T, turning; R, red ripe. E, Immunolabeled with LM19, which labelled de-methyl-esterified HG. Top row micrographs indicate Sepal side of pericarp (Se), middle row micrographs indicate Middle of pericarp (Mi), and bottom row micrographs indicate Stigma side of pericarp (St).

In skin, the pectin content and degree of pectin methyl-esterification was distinctly changed compared with other tissues. The anatomical features of the epidermal cell layers were examined by light microscopy (Fig. 3C). Pericarp tissue slices for each ripening stage were made, with a focus between the skin and mesocarp. These were stained by Ruthenium Red with or without NaOH treatment. These slices were also stained by Toluidine Blue as a control. Whole pectin content increased remarkably from the I to B stages (Fig. 3C-f,g,h), while de-methyl-esterified pectin increased from the B to R stages (Fig. 3C-m,n,o). During fruit ripening, pericarp tissues soften gradually and cell adhesion may weaken. Pectin de-methyl-esterification is probably related to cell–cell adhesion. Moreover, in this study, we have succeeded in observing a whole fruit section of red ripe tomato fruit, including liquefied locular tissue using a devise microscopic fixing techniques (Fig. 7). Those sections were used for the determination of wall modifications at the cellular level by comparing histochemical staining patterns and immunolocalization patterns using antibodies. Immunolocalization of Homogalacturonan (HG) epitopes raised against de-methyl-esterified pectin (LM19) and methyl-esterified pectin (LM20) residues in tomato fruit (sectionhttp://www.plantprobes.net/index.php). Especially, pectin were rich in pericarp than locular tissue (Fig. 7C and D), and methyl-esterified pectin content increased remarkably from the Immature green to Mature green stages (Fig. 7D), while de-methyl-esterified pectin increased from the Breaker to Turing stages (Fig. 7C). Even in the red ripe stage, pectin residues remained in the outline around the pericarp form.

## Discussion

### Differential Control of Methyl-esterification of Pectin is Present in Fruit Tissues during Ripening

Fruit softening is a prominent character of climacteric fruits. Softening of fruit occurs due to solubilisation and depolymerization of cell wall hemicelluloses and pectin by various cell wall hydrolases [9], [30]. Disassembly of the fruit cell wall is largely responsible for softening and textural changes during ripening, but the precise roles of particular cell wall alterations and of the cell wall-modifying enzymes responsible for these changes are unknown. Most studies have focused on the fruit as an organ or only on pericarp softening. The changes during fruit ripening, including seed development, may differ between the pericarp tissues and the inner tissues, locular tissue and placenta (Fig. 1). Therefore, in our study, we compared different tomato fruit tissues and suggest that changes in pectin during fruit ripening differ in each tissue and that pectin is not only degraded but also biosynthesised (Fig. 2).

Pectin sugar composition and construction vary with the different roles typical of tissues and cell layers. Tomato fruits are rich in HG composed of long chains of (1→4) a-d-galacturonic acid, which is initially highly methyl-esterified, and the main chains, polygalacturonans, are secreted into the cell wall in a highly methyl-esterified form, which are de-esterified during cell development. During fruit ripening in tomatoes, the degree of methyl esterification of pectin decreased from 90% in mature green fruit to 35% in red ripe fruit [31]. Although our data were similar to these results, pectin de-methyl-esterification was specific to the pericarp, especially in the cell layers between the skin and mesocarp (Fig. 3C). In contrast, the degree of pectin methyl-esterification in locular tissue was constant at about 50% (Fig. 3B). This was accomplished by pectin methyl esterase (PE; EC 3.1.1.11) and the degree of methyl-esterified pectin was associated with PE activity in all tissues. Demethylation of pectin to their free carboxyl groups changes the pH in the cell wall, allowing for the aggregation of polyuronides into a Ca-linked gel structure, making the polyuronides susceptible to degradation by PG [31], [32], [33], [34], [35]. From these results, changes in the degree of pectin methyl-esterification appear to play an important role in tissue-specific changes in pectin construction during tomato fruit ripening.

Compared to other tissues, the degree of methyl-esterified pectin in locular tissue was low (45–50%) at the immature fruit developing stage (Fig. 3B). This suggests that PE was active early in fruit development or fruit set and pectin was already de-methyl-esterified at these stages. The pectin content increased in locular tissue during ripening; maintaining the degree of methyl-esterified pectin at 50% from the early fruit developing stage is difficult. HG is thought to be synthesised in the *cis*-Golgi, methyl-esterified in the medial-Golgi, substituted in the *trans*-Golgi and secreted in a highly methyl-esterified state [36], [37], [38]. HG GAlactUronosyl Transferase (GAUT) and a Pectin Methyl-Transferase (PMT), probably acting as a hetero-complex, could be involved in the polymerisation of a fully methyl-esterified HG (i.e. 80%), which is the secreted form. Recently, a Golgi-localized HG GAUT was identified in Arabidopsis [23]. Golgi pectin methyltransferase activity was demonstrated [39], [40], and a candidate gene has been identified [41]. In the pectin HG biosynthesis process, the degree of methyl-transferisation differs between tissues (Fig. 3B). The degree of pectin methyl-esterification is especially important to embryonic development in locular tissue and may be associated with seed development.

### Ca-binding Pectin and Hairy Pectin in Skin Cell Layers are Important for Intercellular and Tissue–tissue Adhesion

During fruit ripening, pectin de-methy-esterification due to PE activity was specific to pericarp tissues (Fig. 3A). In skin, although pectin was de-methyl-esterified similarly to mesocarp and pectin was gradually degraded by PG activity (Fig. 5), pectin in the cell wall content was higher than in other tissues and Ca content was 1.5–2 times that in other pericarp tissues (Fig. 4A). The cell wall is the largest pool of Ca^2+^ in plant tissues, reaching about 60–75% of the total tissue Ca^2+^ content [42]. In the cell wall, PEs carry out block-wise de-esterification, creating contiguous stretches of galacturonic acid residues [43]. The extent and strength of Ca^2+^ cross-linking depends on the pattern of de-esterification as well as on the number and availability of the acidic residues [44]. Under low degrees of block-wise de-esterification, pectins associate ionically with carboxyl moieties participating in labile binding with free Ca^2+^, forming plastic gels with low shear strength [45]. As pectins are de-esterified block-wise by PE, dimers begin to form in a cooperative fashion, so that binding strength increases rapidly as the ratio of Ca^2+^ to available binding sites increases [44]. The number of consecutive de-esterified galacturonic acid residues required to form stable chains in a modified, or shifted “egg-box”, configuration [46], [47] has been estimated in various systems to range from 6 to 20 [48]. De-methyl-esterified pectin, which can be cross-linked with Ca, is rich in the skin cell wall, and these pectin residues may form an “egg-box” configuration and pectin gels. Because a great difference in pectin construction exists between the skin and mesocarp, tissue firmness also differs, and these differences may be responsible for the phenomena seen due to the differences in the skin and mesocarp, e.g. skin peeling. The colourless and non-ripening Cnr mutations are recessive and dominant mutations, respectively, and effectively block the ripening process. This could be due to failure to produce elevated ethylene or to respond to exogenous ethylene during ripening [28], [49], [50]. This indicates that high-level cell separation in Cnr is attributable to thin cell walls, weak cell adhesion and fewer pectin–Ca^2+^ bonds. These results suggest that pectin–Ca^2+^ bonding is required for cell–cell or between tissue adhesion, such as between the skin and mesocarp. Also, BER in tomato fruit (*Solanum lycopersicum*) is believed to be a Ca^2+^-deficiency disorder. Consequently, changes in the expression of enzymes that create binding sites for Ca^2+^ in the cell wall, such as PEs, can potentially affect cellular Ca^2+^ partitioning and distribution (Fig. 3B,C). Accordingly, our results show that pectin de-methyl-esterification due to PE and pectin–Ca^2+^ binding exists in cell layers under the skin cuticle layer, as occurs in BER. Our results show that Rha, Ara and Xyl are increased in skin cell walls from the late ripening stage. Pectin is thought to be increased in side chains during ripening in the skin (Fig. 6D). These Ara side chains of pectins may prevent the formation of cross-links between pectins, but some reports showed that Ara side chains are important for the formation of other cell wall networks [51], [52], [53]. The skin is the border tissue between the outside environment, with a cuticle layer on the surface. High-density net construction of pectin and other cell wall components in the skin cell layers under the cuticle seem to be important in determining firmness in fruit softening. Recently, Ara side chains of pectin in RG I were reported to interact with cellulose fibers [53]. Covalent bonding exists between pectin and xyloglucans [54], [55] and pectins are also cross-linked with cell wall proteins, as in extensin [56]. These results suggest that these cell wall net constructions have weak interactions or close covalent bonds, giving these cell walls a supermolecular structure. High-density complex cell wall structures in the skin might be important for maintaining tissue firmness. The cuticle also has a role in resistance to fungal infection and apparently provides resistance to postharvest pathogens. Similarly, the cell wall of the inner skin cell layer under the cuticle is a high-density structure due to increases in pectin side-chains and Ca bonds (Fig. 3C and 6D). These may play a role in resistance to pathogen infection by restricting the size or molecular weight of objects that can pass through the skin.

### Maintenance of Globular Form and Softening of Tomato Fruit are Regulated by the Arrangement of Pectin Structures in Tissues

Micro tom is a miniature dwarf tomato cultivar that was originally bred for home gardening. This cultivar has several unique features, such as a small size that enables it to grow at a high density seed setting under fluorescent light and a short life cycle that allows for mature fruit to be harvested within 70–90 d after sowing. And the genome was fully opened. These features are similar to those of Arabidopsis; consequently, this tomato is considered to be a model cultivar for tomato research. Although Micro Tom has some mutations, for example brassinosteroid deficient phenotype, plant growth and development of Micro Tom appear largely normal compared with more typical tomato cultivars. Most of the information on softening in fleshy fruit ripening is based on studies on tomatoes, and many of these studies suggest that tomato fruit softening is associated with changes in sugar metabolism and proteins affecting the integrity of the middle lamella, which controls cell–cell adhesion and thus influences fruit texture. The degree of pectin methyl-esterification is reduced by PE, and if PE is suppressed, then polyuronide depolymerization decreases [57]. Physical restrictions to PG activity may exist. Although most studies have focused on fruit softening due to pectin de-methyl esterification by PE and depolymerization by PG, experiments with transgenic tomato fruits whose genes encoding these and other wall remodeling proteins had been silenced do not support this hypothesis; these experiments suggest that individual gene knockdowns have small effects on fruit softening [29]. During fruit ripening, cell wall pectin biosynthesis and assembly occurs, and pectins secreted to the apoplast are highly esterified and later de-esterified by the activity of PEs [43], [58] inducing pectin–Ca^2+^ cross-linking, which has an important role in the fruit cell wall. These processes are controlled differently in each tissue. We considered that the degree of softening or firmness is also regulated differently in each tissue.

Pectin de-methyl-esterification by PE is specific in pericarp tissues and skin (Fig. 3A and B), with mesocarp being very similar, but changes in tissue texture differ remarkably between skin and mesocarp. In skin, early ripening stage (MG) cell expansion is stopped and the surface becomes glossy, with the cuticle layer and cell wall becoming thick (Fig. 3C). The expression level of the tomato PMEU1 gene, which encodes the PME1 isoform, is elevated in the mature green stage, with levels declining substantially at the onset of ripening [59]. Gene silencing experiments demonstrate that the loss of PMEU1 expression leads to an enhanced rate of softening during ripening, suggesting that the action of PME contributes to the maintenance of fruit firmness. Rich pectin–Ca2+ cross-linking and the interaction of cell wall components likely produce a high-density cell wall structure that plays a role in maintaining tissue firmness. In the mesocarp, cell wall components are fewer than in the skin (Fig. 6A), and the degree of pectin in the cell wall decreases gradually from the breaker stage (Fig. 6C), which is earlier than in skin. Pectin degradation is associated with fruit softening, and it is thought to increase in the inner fruit tissues, mesocarp, endocarp and locular tissue, with cell adhesion and tissue firmness decreasing during fruit ripening. In mesocarp, pectin de-methyl-esterified occurs, but pectin content, especially pectin–Ca^2+^, is about five times lower than in the skin (Fig. 6B). Pectin is not believed to form a high-molecular-weight polymer or gel in these tissues and is easily degraded by PG, which has a much higher activity than in the skin. Similar to many studies, pectin degradation due to the lack of side chains to interact with other components resulted in weak cell adhesion and decreased cell wall or tissue firmness affecting fruit softening. From the results of immunofluorescent antibody staining of LM19, de-methyl-esterified pectin is present in higher amounts on the calyx side than the stigma side, and increases until the breaker stage and decreases thereafter (Fig. 7C). This indicates that fruit softening starts on the stigma side. Pectin is lower on the stigma side than the calyx side and during ripening, pectin de-methyl-esterification and degradation weaken cell adhesion from the stigma side. At the red ripe stage, pectin content does not differ between the calyx side and stigma side (Fig. 7C and D).

In the septum, which connects the pericarp and placenta, and the separated locules, the cell wall pectin content increases slowly until the breaker stage, remaining stable afterward (Fig. 6A and B). From the gene expression analysis, both pectin biosynthesis and degradation related genes were expressed constitutively, and pectin or cell wall components were reconstructed actively in the septum (Fig. 2A, B and D). High PG activity, especially after the Turning stage was observed (Fig. 5), and although the inner skin-like cell layers, which formed a thin membranous layer outlining the septum, was maintained, the inner septum cell layers gradually became thin. This suggests that both cell wall biosynthesis and degradation actively occurs, and decreasing septum firmness affects fruit softening. PG activity also increased from earlier ripening stages compared to other tissues, and softening may start with decreasing septum firmness.

Locular tissue is a major expanding tissue in tomato fruit. The degree of pectin methyl-esterification in this tissue was constant at about 50% (Fig. 3B). Changes in bound Ca are associated with increasing pectin content. Although the pectin in locular tissue also can be cross-linked with Ca in the cell wall, pectin content was remarkably low and finding bound pectin-Ca or gel was difficult. In contrast, PG activity was high in later ripening stages as in pericarp tissues as pectins were degraded and decreased (Fig. 6B). These results indicate that pectin methyl-esterification levels vary among specific tissues and cause diverse modifications in the degree of cell wall degradation during fruit development.

In tomato fruits, fruit drop or birds disperse seeds. Globular form of fruit might be maintained by the skin and septum until seed maturity when seed surrounding tissues (mesocarp, endocarp and locular tissue) become soft, allowing for easy seed drop. Each tissue, according to its role in fruit ripening, seems to be differentially controlled in fruit cell wall modification and construction.

## Supporting Information

File S1 **Contains Figure S1 and Table S1**: Figure S1 Alignment of GAUT1-like family. Amino acid sequences of GAUT1-like family from Solanum lycopersicum (SGN-U565384, SGN-U565385, SGN-U598345) and GAUT1 from Arabidopsis thaliana (AT3G61130) were aligned using GANETIX. Table S1 The primer pairs for pectin biosynthesis and depolymerization-related gene expression analysis. The gene expression was analysed by RT-PCR using these primers (Fig. 2). PE2 (Fig.2A), PG2(Fig.2B) and GAUT1-like family (Fig.2C) genes expression were compared to rRNA(Fig. 2D) in the same assay.
(DOCX)
