# Supplementary material for: Tissue Specific Localization of Pectin–Ca2+ Cross-Linkages and Pectin Methyl-Esterification during Fruit Ripening in Tomato (Solanum lycopersicum)
Source: PLoS One. 2013 Nov 13;8(11):e78949. doi: 10.1371/journal.pone.0078949 (PMC3827314; doi:10.1371/journal.pone.0078949)
Supplement: File S1 — Contains Figure S1 and Table S1: Figure S1 Alignment of GAUT1-like family. Amino acid sequences of GAUT1-like family from Solanum lycopersicum (SGN-U565384, SGN-U565385, SGN-U598345) and GAUT1 from Arabidopsis thaliana (AT3G61130) were aligned using GANETIX. Table S1 The primer pairs for pectin biosynthesis and depolymerization-related gene expression analysis. The gene expression was analysed by RT-PCR using these primers (Fig. 2). PE2 (Fig.2A), PG2(Fig.2B) and GAUT1-like family (Fig.2C) genes expression were compared to rRNA(Fig. 2D) in the same assay. (DOCX) [file pone.0078949.s001.docx]

**Supplementary Information**

Figure S1. Alignment of GAUT1-like family.


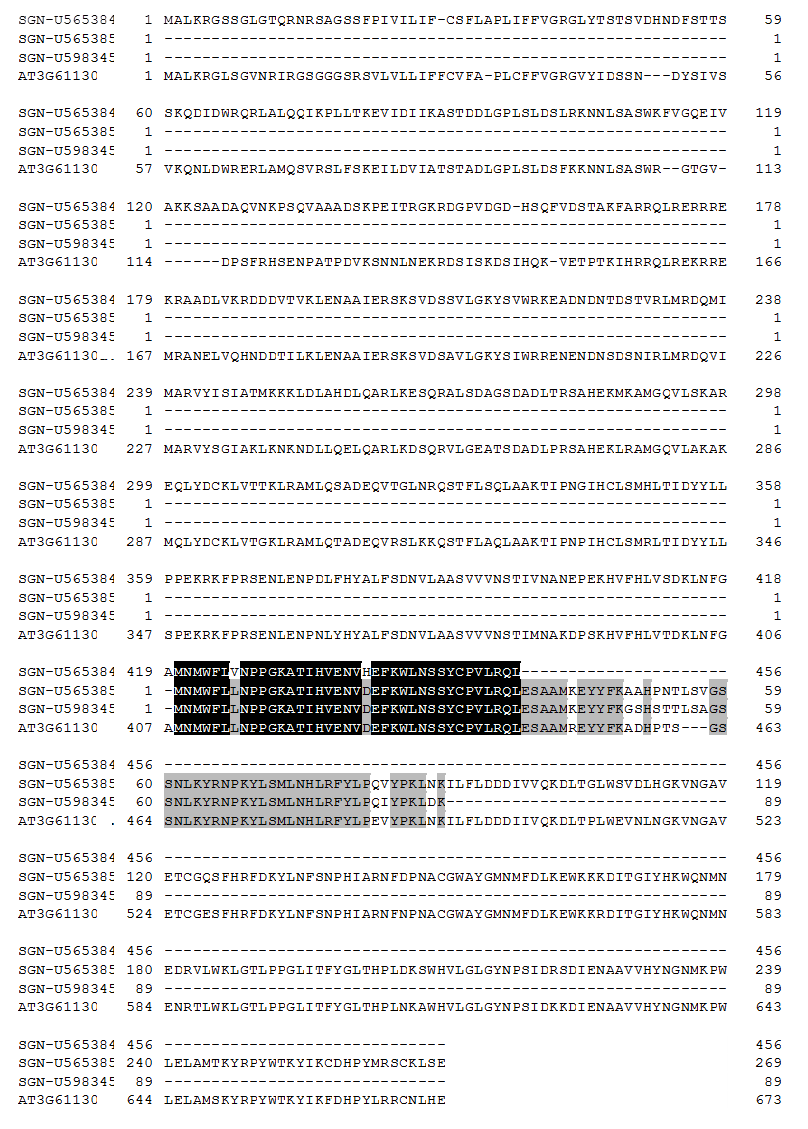


Table S1. The primer pairs for pectin biosynthesis and depolymerization-related gene expression analysis.

|  | **FORWARD PRIMER** | **REVERSE PRIMER** |
| --- | --- | --- |
| **PE2** | ATACAGAACACAGCAGGACCAGC | AGGCCACTTGACACGCTTACTAG |
| **PG2** | GAGCCCAAATACTGATGGAG | GATCCTCCCTGCCAAGTCTTGAT |
| **GAUT1-like family** | TCTTCGCTCTTAGACCTTTTAGG | CATCAAGAAAAAGGATTTTATTCA |
| **rRNA** | GCAAATTACCCAATCCTGAC | CTATTGGAGCTGGAATTACC |
